# Predicting Injury in Collegiate Baseball and Softball Athletes Using Functional Testing: A Pilot Study

**DOI:** 10.3390/muscles4020010

**Published:** 2025-04-09

**Authors:** Alyse M. DePaola, Andrew R. Moore, Graeme J. Connolly, A. Maleah Holland-Winkler

**Affiliations:** Department of Kinesiology, Augusta University, Augusta, GA 30909, USA; 22.alyse@gmail.com (A.M.D.); andmoore@augusta.edu (A.R.M.); gconnolly@augusta.edu (G.J.C.)

**Keywords:** athletic injuries, musculoskeletal system, functional movement screen, closed kinetic chain, pre-season testing, injury prediction

## Abstract

Non-contact injuries are common in collegiate throwing athletes. Identifying musculoskeletal issues that predispose athletes to injuries would be valuable for reducing the associated risk. The purpose of this pilot study was to use binomial logistic regression to identify injury-prone athletes with multiple pre-season functional measures and demographic information. Eighteen Division II baseball and softball athletes underwent pre-season functional testing including measures of manual muscle testing of the dominant shoulder muscles (MMT), the functional movement screen (FMS), and closed kinetic chain upper extremity stability (CKCUES). A certified athletic trainer at the university diagnosed and documented the injuries that these athletes sustained over the course of the season. Binomial logistic regression models were used to assess the effects of FMS composite score, CKCUES normative score, MMT scores, and demographic information on the likelihood that participants would sustain (a) any type of injury and (b) a shoulder injury during the competitive season. The model for injury was not significant (*p* = 0.822), correctly classifying 72.2% of cases. The model for shoulder injury was significant (*p* = 0.039) and correctly classified 100% of cases. These results suggest that shoulder injury incidence may potentially be predicted using sport-specific movement tests in baseball and softball athletes. A larger sample size is needed to verify these results in the future.

## 1. Introduction

Musculoskeletal injuries are frequent in collegiate athletes. Approximately 11,000 injuries occur each year in NCAA-sanctioned sports [1]. These injuries range in severity and amount of lost participation time, total recovery time, and medical resources required for their care. Some injuries, particularly those that require surgery, can cause future difficulties. Interestingly, non-contact injuries account for 17.7% of injuries during games and 36.8% of injuries that occur during practice [2]. These numbers may not be significant, but non-contact injuries have been of particular interest among healthcare professionals. Most injuries sustained are contact injuries; however, not much can be done to prevent contact injuries. Non-contact injuries are worth examining because researchers have identified certain risk factors for various injuries. Some risk factors are anatomical and cannot be remedied without surgery; however, there are biomechanical abnormalities that can predispose an individual to injury. These abnormalities can be identified through assessing movement patterns, asymmetries, or balance abnormalities [3].

If such abnormal biomechanical patterns can be linked to injury predisposition, sports medicine personnel can incorporate such tests into pre-season screening. To date, pre-season physical exams have been effective at identifying athletes with current injuries but have not been able to be a predictor of future injury [4]. Similarly, preparticipation examinations have long been used to assess an individual’s ability to safely participate in physical activity at the time of examination, but no existing screening test has been shown to predict an individual’s risk of injury when participating in future activities. Therefore, if functional movement and muscle testing can be useful in identifying those at risk of injury, these tests can be incorporated into the traditional pre-season physical exams. Once identified, these factors can be modified or corrected to decrease an athlete’s chance of injury. For instance, a meta-analysis of randomized controlled trials by Lauersen et al. (2014) reviewed 25 trials and demonstrated that strength training significantly reduced sport and overuse injuries [5].

Collegiate baseball and softball are ranked among the lowest for injury rates in practices and games. However, the types of injuries are of more concern than frequency with these particular athletes. Overuse upper extremity injuries are common amongst overhead athletes [6]. Overuse injuries are a result of repetitive movement patterns without adequate recovery. Therefore, baseball and softball players are susceptible to upper extremity overuse injuries due to throwing repeatedly for long periods.

Throwing mechanics involve a series of motions throughout the entire body that culminate with the release of the ball. Forces are generated from the ground up in what is known as the kinetic chain. The kinetic chain refers to the linkage of multiple body segments that allows for the transfer of forces and motion [7]. The kinetic chain can be very complex to analyze. The kinetic chain requires optimal anatomy, physiology, and mechanics to transfer energy efficiently and effectively from the legs and core to the arms. Therefore, breakdowns that occur anywhere within the kinetic chain can lead to injury. These impairments include altered motor control, muscle strength, flexibility, endurance, joint injury, and improper muscle activation patterns [6].

The functional movement screen (FMS) is a screening test developed to identify deficits in movements that may predispose an otherwise healthy individual to injuries during activity [8,9,10]. More specifically, its purposes are to (1) identify body asymmetries, (2) assess mobility and stability within the kinetic chain of whole-body movements, and (3) detect poor-quality movement patterns [8,10]. Applications of the FMS include establishing a baseline for movement proficiency to allow for comparisons after performance training, treatment, and rehabilitation, as well as screening for the possibility of future injury.

One of the first groups to investigate the possible predictive value of the FMS was Kiesel and colleagues (2007), who found that lower scores predicted a significantly higher injury risk in professional football players [11]. As a result of these initial findings and the potential utility of such a screening test, the FMS was widely adopted in organizations such as the National Hockey League (NHL) and the National Football League [11,12,13]. More recent studies involving the FMS, however, have produced varied results regarding the injury-predictive value of the test [1,9,14,15,16]. Indeed, there are researchers who have reported inconclusive FMS scores when it comes to predicting those who are prone to injury [3,17,18]. Furthermore, Dorrel and colleagues (2018) found that the FMS alone has only a slightly better than 50/50 chance of accurately identifying athletes at risk of future musculoskeletal injuries [16]. The FMS has also been shown to have excellent inter-rater and intra-rater reliability, although significant concerns remain regarding the validity of the FMS [19].

Although not its intended use, the FMS has been utilized in various studies to try to identify a score that correlates with one sustaining an injury. The goal when developing the FMS was to identify weak points and subsequently develop corrective exercise programs based on the observations [20]. Garrison, Westrick, Johnson, and Benenson (2015) found that a score of less than 14 combined with a history of injury increased one’s chance of injury by 15 times [21]. Another study also concluded that lower FMS scores correlated with injury in female Division II athletes [14].

In addition, a history of previous injury may affect one’s performance on the FMS. Specifically, athletes that had a history of hip, hand, elbow, or shoulder injury had lower FMS scores [3]. Therefore, it should be expected that those with a previous injury history should have lower scores. Contradictory to what those studies found, Bardenett et al. (2015) did not find specific FMS scores to correlate with high school athletes that were more injury-prone [17]. Their research suggests that the FMS may be unreliable in the high-school-aged population. Warren et al. also did not find FMS scores to be indicative of future injury in various Division I sports [1,3]. Therefore, it is inconclusive if FMS can predict future injury in team-sport athletes. Additionally, most studies analyze multiple sports, with very few being focused on one specific sport.

The incorporation of several screening tools into a battery of functional testing may be a more accurate assessment technique for identifying injury-prone athletes. The results of individual tests are susceptible to bias and measurement errors, resulting in highly variable levels of inter- and intra-rater reliability (see Bonazza et al. 2016 for review) [19]. Including tests that are specific to the types of movements commonly encountered by the athlete would further increase the meaningfulness of the results. Athletes in sports that are characterized by overhead throwing, such as baseball and softball, are at a particularly higher risk for non-contact overuse injuries due to a relatively greater number of competitive events per season and the near-maximal levels of force production during throwing activities.

Some research to date supports the concept of using a variety of tests to predict injury outcomes in these types of athletes. However, these studies have limited generalizability to all throwing athletes [22] or focus more on subjective symptoms of injury rather than diagnosis by trained practitioners [4]. A series of tests that is applicable to a wider range of throwing athletes, including females, would provide more opportunities to diagnose and correct for functional impairments related to injury. Currently, no consistent tests are performed during pre-season to detect injuries. Thus, the current study aimed to determine the accuracy of a regression model for predicting injury incidence in throwing athletes using the FMS, related functional test scores, and a self-report questionnaire as predictor variables. If at-risk athletes can be identified through this testing, then those athletes can undergo therapeutic rehabilitation interventions to correct or modify the identified risk factors.

## 2. Materials and Methods

### 2.1. Experimental Design

**Figure 1:**
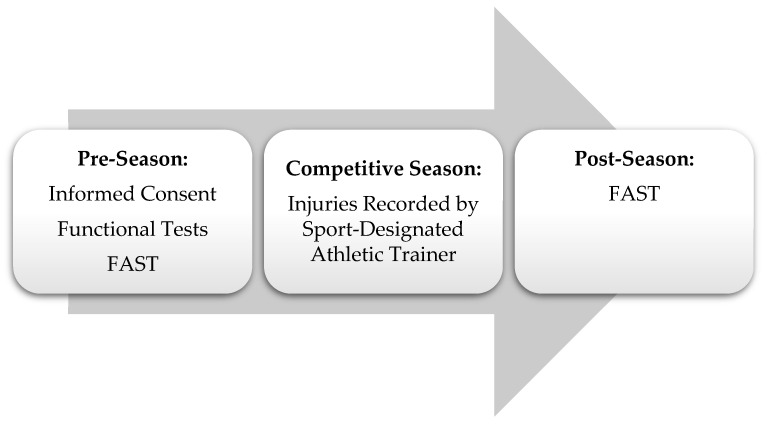
Order of measures during pre-season, competitive season, and immediately after competitive season.

This exploratory study analyzed the accuracy of a regression model to determine if functional movement test scores may play a role in predicting injury during the competitive season in Division II baseball and softball players. The participants visited the laboratory one time during the first month of pre-season, which included signing an informed consent form and completing multiple functional tests. Data collection during the laboratory visit started with completing the Functional Arm Scale for Throwers questionnaire (FAST). Following completion of the FAST, height and weight were recorded, followed by functional tests. The functional tests started with manual muscle testing (MMT) of the upper extremity and core musculature. After MMT, functional movement screen (FMS) testing was completed. The final portion of the laboratory visit involved completing the closed kinetic chain upper extremity stability (CKCUES) test. An athletic trainer then recorded injuries sustained throughout the competitive season. Participants completed the FAST again after the competitive season to provide subjective data on throwing arm pain to provide changes from pre-season to post-season. The testing order is displayed in Figure 1. The university’s Institutional Review Board approved this study, and all procedures performed followed institutional guidelines.

### 2.2. Participants

Eighteen Division II baseball and softball athletes completed the study. The inclusion criteria for participation included being a collegiate baseball or softball team member. Athletes with a current injury preventing participation and pregnant athletes were excluded. Of the 18 participants, 11 were female and 7 were male. Participant characteristics are summarized in Table 1. Players of all positions were assessed. These 18 participants completed all facets of the research design and, thus, were all included in the statistical analysis.

### 2.3. Procedures

Participant height and weight were assessed using a stadiometer and scale (Continental Scale Corp., Bridgeview, IL, USA). Testing began when the participants filled out the FAST, which was completed at the pre-season laboratory visit and at the end of the competitive season. The FAST is an upper-extremity-specific patient-reported outcome designed to assess the impact of upper extremity disorders in high-demand throwing athletes [23]. The questionnaire contains 22 questions, both sport-related and non-sport-related, graded on a Likert scale. The questions fall into five domains: pain, throwing, activities of daily living, psychological impact, and advancement [23]. The score obtained is converted to a scale ranging from 0 to 100, such that higher scores indicate a lower health-related quality of life. In an analysis of five studies including injured and uninjured throwing athletes, the FAST was found to be a reliable, valid, and responsive upper extremity region-specific and population-specific patient-reported outcome scale [24]. In the current study, the changes in FAST were evaluated to characterize the level of stress and change in upper extremity health-related quality of life reported as a result of completing the competitive season.

Next, MMT was performed to measure the force of motion in different planes of the upper extremity and core musculature using a handheld dynamometer (Lafayette Manual Muscle Tester, Lafayette, IN, USA). The shoulder was assessed bilaterally for strength of flexion, abduction, and extension. The participants lay supine on an examination table for the assessment of flexion and abduction, and then prone for the extension assessment. The dynamometer was placed on the middle portion of the upper arm, distal to the deltoid insertion. The participant was instructed to push as hard as they could in the specified direction. Each plane was assessed three times, and the average score was recorded.

After MMT, FMS testing was performed. The FMS is a system of observing seven functional movement patterns to identify dysfunction within basic movement patterns. The same order of tests as described in the FMS manual was used for all participants, starting with a deep squat, and then assessing a hurdle step, linear lunge, shoulder mobility, an active leg raise, a trunk stability push-up, and rotary stability. Each functional movement was first demonstrated to the participant by the investigator. How to perform each of the tests is described in detail in the FMS manual [20]. The scores were recorded. For the tests performed bilaterally, the lower of the two scores was recorded, as per the scoring criteria. For the FMS, each movement is observed and scored on a scale from 0 to 3, making the maximum composite score 21. A score of 0 on any of the tests indicates pain with the movement or the inability of the participant to perform the movement. Conversely, a score of 3 indicates that the participant performed the movement perfectly. A score of 2 indicates some modification, and multiple modifications yields a score of 1 [20]. For the movements performed bilaterally, the lower of the two scores was the score utilized to calculate the composite score.

Lastly, participants completed the CKCUES test. The CKCUES test requires the participant to be in a push up position with their hands placed 36” apart. Tape was placed on the ground to indicate hand placement for the participants. Once positioned, the participants had 15 s to touch their supporting hand with their swinging hand as many times as possible. To perform the test correctly, the participant must keep their knees up, keep their upper extremity perpendicular to the floor and over their hands, and maintain their feet in their initial position. If needed, the participant can perform the test from their knees [25]. The test was repeated twice, with 45 s of rest in between sets. The greatest number of touches between sets was the final score recorded. The proper positioning and protocol were first demonstrated and explained to each participant prior to starting. The CKCUES test has demonstrated high validity when correlated with maximum grip strength and peak torque of internal/external shoulder rotation [26]. The mean reference values for adults between the ages of 18–25 are 18.68 touches for women and 18.98 touches for men [27].

Over the course of the season, the athletic trainers specific to each team evaluated and documented any injuries sustained. All injury data were documented in the Athletic Trainer System (ATS) online program. This program was only accessible by the athletic trainers at the university. The documentation included the type of injury, injury severity, date sustained, and date of return to play, as well as other pertinent information. These data were collected throughout the regular season in the ATS, and injuries were also added to each participant’s file.

The investigator who conducted and evaluated the pre-season functional tests was an athletic trainer. Different athletic trainers, who were specifically paired with the baseball and softball teams and not investigators in this study, reported the injuries. The data analysis was performed by an investigator who was not involved in evaluating the functional tests or the diagnosis of injuries.

### 2.4. Statistical Analysis

All data were processed using the statistical software program SPSS, version 29 (IBM, Armonk, NY, USA), with the alpha level set to 0.05. A paired-samples *t*-test was used to compare the FAST scores taken during the pre-season and post-season. The differences in FAST scores for each participant at these time points were screened for outliers by assessing standard scores (values > 3 SD units from the average) and for normality using normal Q-Q plots (visual assessment). Effect size was reported as Cohen’s d.

Two binomial logistic regression analyses were performed to determine the effects of relevant muscular ability and demographic variables on the likelihood of injury in study participants. One analysis was for all types of injury, and the second analysis was specific to shoulder injuries. The Box–Tidwell procedure was used to test for the assumption of linearity. The residuals of scores were screened to identify outliers (values >3 SD units from the average). The explained variance in the outcome measure for each model is reported as Nagelkerke R^2^. Classification charts were constructed to show the sensitivity and specificity of each model in predicting their specified outcomes. Receiver Operating Characteristic (ROC) curves were generated to visually represent the discrimination ability of each model.

## 3. Results

Out of the 18 participants included in the study, a total of 9 participants sustained an injury over the course of the season. Of those 9 injuries, all were non-contact, and only 3 were shoulder-related. The results of the tests and the demographic information for all categories of athletes (injured, shoulder injury, not injured, and all participants) can be found in Table 1. The raw data and resulting statistical analysis files can be accessed at the following external data repository: https://osf.io/v8pbt/ (accessed on 28 March 2025).

Two binomial logistic regression analyses were performed to predict the likelihood that this sample of 18 athletes would sustain the following outcomes: (a) an injury of any kind and (b) specifically a shoulder injury. The predictor variables used in both analyses included height, weight, sex, FMS composite score, closed kinetic chain upper extremity stability (CKCUES) normative score, and manual muscle testing (MMT) data for the dominant shoulder. The data met the assumption of linearity (*p* > 0.05 in both cases) and no outliers were detected.

The logistic regression model for any kind of injury was not statistically significant: χ^2^(8) = 4.38; *p* = 0.822. The model explained 28.8% of the variance in injury and correctly classified 72.2% cases. The logistic regression model was statistically significant for predicting shoulder injury: χ^2^(8) = 16.22; *p* = 0.039. The model explained 100% of the variance in injury and correctly classified all cases. None of the eight predictor variables in either of the regression models were statistically significant. The classification chart information for the two binomial logistic regression models is depicted in Table 2.

**Figure 2:**
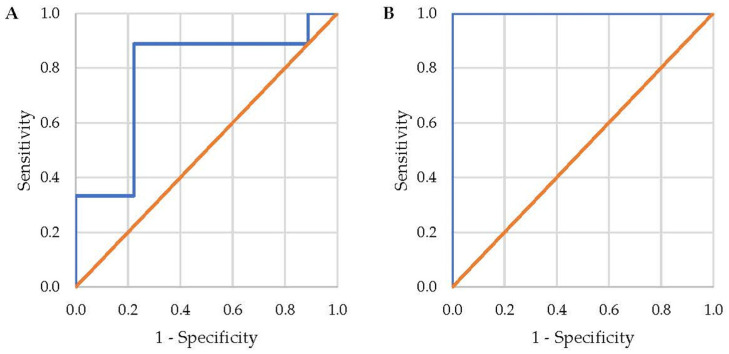
ROC curves for binomial logistic regression models predicting (**A**) all injury types and (**B**) shoulder injuries. Each figure consists of the ROC curve based on the regression model (blue line) and the no-discrimination (orange) line which represents the curve that would be generated from a random guess model.

The area under the ROC curve (AUC) for the injury model was 0.778 (95% CI, 0.543–1.000) which is considered an acceptable level of discrimination. The AUC was 1.000 (95% CI, 1.000–1.000) for the shoulder injury model, since all cases were correctly identified [28]. The ROC curves for each mode are presented in Figure 2.

## 4. Discussion

The purpose of this study was to determine the accuracy of a regression model for predicting injury incidence in collegiate throwing athletes using a variety of diagnostic and screening tools related to functional movement and strength. This model, which utilized sex, height, weight, FMS composite score, CKCUES normative score, and MMT scores of dominant shoulders, was able to predict 72.2% of overall injury occurrence and 100% of shoulder-specific injury occurrence during the competitive season in baseball and softball athletes. The injuries sustained were all non-contact injuries and included muscle strains, ligamentous injuries, and tendonitis; specifically, there were two cases of biceps tendinitis, one case of supraspinatus tendinitis, two cases of quadricep strain, three cases of ankle sprains, and one ACL tear. Those who sustained an upper extremity injury were pitchers. The nature of being a pitcher makes these individuals more prone to overuse upper extremity injury.

There has been a lot of discrepancy in the literature as to whether the FMS alone can identify injury-prone athletes. The FMS is a great tool to identify abnormalities in movement, and some have tried to correlate these deficiencies with injury risk. The included tests identify weak points in three categories: mobility, motor control, and functional patterning [20]. However, the deficiencies identified through the FMS are not sport-specific, but rather inherent to sport movement [18]. Therefore, more sport-specific testing or analysis needs to be conducted to properly identify injury cause and then those who are more injury-prone. In a systematic review by Moran, Schneiders, Mason, and Sullivan, (2017), it was concluded that the association between the FMS and injury was not enough to recommend its use as an injury-prevention tool [29]. These conclusions were made while looking at multiple sports at once, as well as with just the use of FMS without additional tests. Other studies have tried to incorporate additional factors such as previous history of injury or other functional testing measures to attempt to identify those who are more injury-prone. The current study attempted to utilize multiple tests to try to identify those more injury-prone athletes. While FMS composite scores were part of the regression model used to potentially predict injury, additional screening tools were incorporated in response to previous literature suggesting the use of multiple tests to increase assessment accuracy and injury prediction potential. In addition to incorporating other tests, the current study was sport-specific in order to reduce variability in determining the usefulness of these tools in potentially predicting injury.

The normative score for the CKCUES test has been utilized in other studies as an injury predictor, and was thus incorporated into this study’s analysis. The CKCUES test can assess one’s performance and functional ability. The CKCUES test was found to be reliable in various populations, including upper extremity sport-specific athletes [25]. Excellent intraclass correlation coefficient (ICC) values were found for both intrasession and intersession reliability [25]. For this study, the number of touches during the CKCUES test ranged from 14 to 32. The reference value for healthy, upper extremity sport-specific males is 18.5 touches [25]. All but one male was above this reference mark. However, the male participants in this study were below the reference marks for the normative scores. The reference values for females are based on those in the kneeling push-up position, rather than the standard push-up position that the participants were in. Therefore, no comparisons can be made between the females in this study and the study by Tucci et al.

The FAST score was not included in the regression model, but the pre- and post-season scores are included in Table 1. The FAST is a valid and reliable measure of a patient’s injury or condition, demonstrating a good test–retest reliability and good-to-excellent concurrent and known-groups validity [24]. The FAST scores for all participants ranged from 22 to 44. A score of 22 correlates with no feeling of pain or disability and a maximum score of 110 correlates with complete disability and extreme pain [24]. The average FAST score prior to the start of the season was 29.7 + 7.0, and post-season it was 34.3 + 11.7. This increase is to be expected, as the demands of the season may have caused increased pain and disability in the throwing shoulder. Additionally, the FAST has a module specific to pitchers. There are only 12 questions involved in this module; therefore, the minimum to maximum scores ranged from 12 to 60. The average scores among pitchers pre-season and post-season were 12.6 + 4.5 and 16.5 + 6.0, respectively. For the overall FAST, some participants had higher scores, some stayed the same, and some even had lower scores from pre- to post-season. Although FAST scores significantly increased from pre-season to the end of the competitive season, the clinical relevance of this is unknown as the scale does not provide severity classifications based on its scores. Instead, it offers general insight into the throwing athlete’s health status over time [23].

The injuries sustained by the participants in this study were non-contact injuries. These injuries are more easily studied because there are identifiable risk factors for non-contact injuries. Contact injuries are difficult to study because they result from uncontrollable and inconsistent factors. The two types of non-contact injuries are acute and chronic; chronic injury is also known as overuse injury. An epidemiology study performed with collegiate athletes found that 29.3% of observed injuries were from overuse [6]. In the current study, three of the nine injuries were overuse-related, yielding similar results to Yang et al.’s findings. Men have been shown to exhibit higher overall rates of shoulder injuries; however, women are more prone to overuse-related shoulder injuries, possibly due to a greater susceptibility to shoulder laxity and rotator cuff tears [30,31]. Following shoulder injuries, women also report poorer functional outcomes, increased pain, and reduced activity levels. The current study had two females and one male sustain an overuse injury. However, there were more female participants than male ones.

According to the model used, injury and shoulder injury have the potential to be correctly predicted. Injury-prone athletes may be identified by utilizing the specified tests. Once identified, the athletic trainer or other healthcare personnel can detect each athlete’s areas that need improvement. For some, it may be mobility, and for others, strength or stability. A comprehensive and individualized plan can be used for each athlete to best suit them. A review by Mayes et al. provides a whole-kinetic-chain-focused training approach to prevent injuries in throwing athletes [32]. However, more research will be needed to conclude if improving one’s score decreases the likelihood of injury.

One major limitation of the findings presented is the small sample size. We used a convenience sample of 18 volunteers from baseball and softball teams in the same season to control for confounding variables such as changes in coaches and athletic trainers. The findings from the underpowered analyses are difficult to generalize to all throwing athletes. A second limitation is the use of a large number of predictor variables to explore the potential for their use in injury prediction models. While the use of many predictors can shed light on their usefulness to this end, the resulting regression models may suffer from overfitting, in which the prediction equations are constructed to suit only the specific sample of cases included, yielding models of questionable generalizability [33]. The cross-validation of a predictive model with novel data can be used to dampen the effects of overfitting, but this process was not feasible with the number of predictors and participants included in this study. It is important to emphasize that the specific models reported in this study are limited in their application, despite providing some evidence that properly constructed predictive models using easily administered and valid functional testing is a possibility worthy of further exploration.

The use of a larger sample of throwing athletes would allow for the inclusion of a wider variety of predictor variables, which is a third limitation we identify here. Although measures of shoulder strength in different planes of movement using MMT were incorporated into this regression analysis, trunk strength was not greatly explored in this study but could have effects on injury risk. Trunk strength can greatly influence one’s throwing motion and the dynamic forces placed upon the joints of the upper extremity. Decreased core musculature can cause a greater reliance on the upper extremity to produce force, which can lead to injury [34,35]. This may serve as a limitation of this study, as the authors did not establish a standard core strength measure in which comparisons could be made. Another limitation to this study was the small sample size. The findings demonstrate the promise and efficacy of using multiple screening tools to identify injury-prone athletes, but additional data collection in a larger sample of athletes will be needed to confirm these results.

Research in this area should also consider the age of athletes when attempting to predict injury likelihood. Our sample was relatively homogenous in terms of age, but professional athletes of a given sport can vary greatly in this regard. Future attempts to predict injury outcomes using screening batteries such as this should consider the effect of age for this reason. Finally, future research should consider the physical demands and stress encountered by athletes of different positions. For example, pitchers—who encounter high volumes of stress more infrequently than positional players—may require a unique model for injury prediction.

## 5. Conclusions

Numerous factors have been identified as risk factors for injury. This study attempted to identify if multiple factors could be utilized together to identify upper extremity athletes at risk for injury. Utilizing FMS composite score, CKCUES normative score, MMT data for dominant shoulders, and demographic information including height, weight, and sex, we were able to accurately identify those that would experience an injury as well as a those who would experience a shoulder injury. This research suggests that injury-prone baseball and softball athletes have the potential to be predicted through pre-season screenings and utilizing logistic regression, as demonstrated in this study.

## Figures and Tables

**Table 1:** Demographic information and test data expressed as mean (M) and standard deviation (SD). Number of females indicated following sample size.

	Injured (*n* = 9; 6f)	Shoulder Injury (*n* = 3; 2f)	Not Injured (*n* = 9; 5f)	All (*n* = 18; 11f)
	M *(SD)*	M *(SD)*	M *(SD)*	M *(SD)*
Height (cm)	172.7 *(10.8)*	173.1 *(8.5)*	173.6 *(13.8)*	173.1 *(12.0)*
Weight (kg)	79.4 *(11.0)*	75.8 *(0.6)*	77.6 *(13.1)*	78.5 *(11.8)*
Shoulder Extension	21.5 *(4.5)*	19.5 *(4.1)*	20.7 *(3.5)*	21.1 *(4.0)*
Shoulder Flexion	24.5 *(5.1)*	22.7 *(1.7)*	25.6 *(3.0)*	25.1 *(4.1)*
Shoulder Abduction	22.7 *(5.7)*	21.5 *(6.6)*	24.2 *(4.5)*	23.5 *(5.1)*
FMSCS	16.0 *(5.2)*	16.0 *(1.7)*	16.6 *(1.8)*	16.3 *(1.6)*
CKCUES Normalized	0.1 *(0.0)*	0.1 *(0.0)*	0.1 *(0.0)*	0.1 *(0.0)*
FAST (Pre-Season)	30.0 *(6.7)*	30.7 *(11.5)*	29.4 *(7.7)*	29.7 *(7.0)*
FAST (Post-Season)	34.0 *(13.7)*	36.3 *(12.9)*	34.7 *(14.5)*	34.3 *(11.7)*

Abbreviations: *n* = number of participants in each category; FMSCS = functional movement screen composite score; CKCUES = closed kinetic chain upper extremity stability test; FAST = functional arm scale for throwers questionnaire. No outliers or violation of normality identified for FAST results, which increased among entire sample from pre-season (29.7 ± 7.0) to post-season (34.3 ± 11.7; *p* = 0.048, d = 0.502).

**Table 2:** Classification chart for injury and shoulder injury models.

		Predicted		
		Injury		% Correct
Observed		No	Yes	
Injury	No	5	4	55.6
	Yes	1	8	88.9
		Shoulder Injury		
Shoulder Injury	No	15	0	100
	Yes	0	3	100

## Data Availability

The raw data and resulting statistical analysis files can be accessed at the following external data repository: https://osf.io/v8pbt/.
